# The Unsung Hero of Neglected Tropical Diseases: Interview with Narcis Kabatereine

**DOI:** 10.1371/journal.pntd.0000546

**Published:** 2009-12-22

**Authors:** Gavin Yamey

**Affiliations:** Public Library of Science, San Francisco, California, United States of America

To reach Dr. Narcis Kabatereine's office at the Vector Control Division (VCD) of Uganda's Ministry of Health in Kampala, you must first walk through a room full of lab technicians who are studiously looking down microscopes.

**Image 1:**
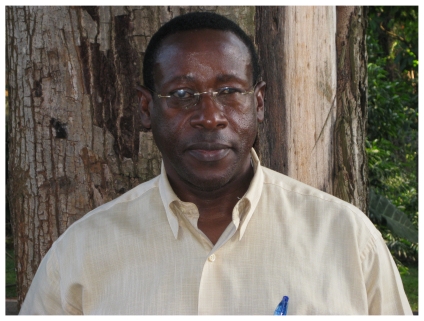
Narcis Kabatereine in Kampala, Uganda (photo by Gavin Yamey).

The atmosphere of quiet diligence is set by the softly spoken Dr. Kabatereine (Image 1) himself, a 56-year-old entomologist, who takes great pride in having trained this cadre of hard-working technicians. When I first meet him, he shows me photos on the bulletin board behind his desk of the young technicians who have died of AIDS—a sobering reminder of the country's death toll. “AIDS hit Uganda hard,” he says. “At one point, every night we were going to a vigil for someone who died.”

Dr. Kabatereine manages the ministry's Bilharzia and Worm Control Programme and chairs the Neglected Tropical Disease (NTD) Secretariat in the VCD. Without fanfare, he has steadily built a career as both an academic and a highly trusted technical expert whose advice on tropical disease control is greatly in demand across Africa. Rwanda, Mozambique, Tanzania, and southern Sudan have all called on him to help guide their NTD control efforts. Dr. Simon Brooker of KEMRI/Wellcome Trust in Nairobi, Kenya, calls him “the unsung hero of NTDs.”

Dr. Kabatereine's office gives a snapshot of his wide-ranging research, consultancies, and advocacy. There are files and papers everywhere. A box on the shelf is labeled “10% Kato Katz Slides,” used in the diagnosis of schistosomiasis. His bulletin board is plastered with newspaper clippings—one striking headline reads “Bilharzia Threatens Hundreds in Uganda.” There's a calendar from the Carter Center. And there are photos on almost every inch of his walls, including one of his PhD graduation ceremony on March 28, 2001, at the Danish Bilharziasis Laboratory (DBL) in Copenhagen.

As a child, he says, “I just loved insects, wondering how the social insects managed to work together—bees, termites and ants.” He followed his fascination at Makerere University in Kampala, where, for his bachelor's degree in zoology and botany, he did a dissertation in entomology while at the same time gaining a diploma in education. During his degree, he says, “my interest in insects increased when I realized that many were vectors of disease.”

That realization led him to join the VCD in 1980. At that time, he says, there were documents showing that schistosomiasis was a problem in north western Uganda “but no one was working on it.” In 1982, he did a 4-month course at the DBL called “Fresh water snails of Africa and schistosomiasis control.” He later did a master's degree in medical entomology at Nairobi University in Kenya, with a focus on tsetse flies and trypanosomiasis control. But when it came to choosing a topic for his doctorate, he says, he chose “snails and schistosomiasis, a disease that was then more neglected than trypanosomiasis.”

Throughout this time, he was becoming steadily more senior at the VCD, eventually becoming the division's Principal Entomologist in 2007. He was devoting much of his life to understanding and controlling Uganda's enormous burden of schistosomiasis and intestinal worms. His studies included the epidemiology of these parasitic diseases in Kampala, and the transmission of *S.mansoni* both in North Western Uganda and in a remote fishing community on Lake Albert.

I ask him to tell me which of his many achievements gives him the greatest pride. “I managed to map out the magnitude of the problem due to schistosomiasis and soil transmitted helminthiasis in Uganda,” he says, “and initiated a national control programme for the diseases.” He also succeeded in including these neglected conditions in the country's Health Sector Strategic Plan. He is proud of having convinced the government and external donors that mass drug administration (MDA) to control these worms was necessary. Through Uganda's MDA campaign, he says, “we have successfully reduced morbidity due to schistosomiasis and soil transmitted helminthiasis.” Since the late stages of schistosomiasis are deadly, he believes the campaign has also saved many lives.

“Appearances with Narcis can be deceptive,” says Dr. Brooker. “To some who do not know him well, he seems happy and grateful to have donors interested in NTD control in Uganda. With time, you realize that long before donors arrived, he had identified what was required to develop an effective and sustainable helminth control programme in Uganda. He then strategically set about to put those pieces in place, identifying which collaborators could support each area.”

Dr. Jan Kolaczinski, Senior Specialist for NTDs at the Malaria Consortium in Kampala, who works closely with Dr. Kabatereine, says that “one of the key character attributes of Narcis is he genuinely wants to do good for his country—he's not a career person.” This attribute is echoed by Dr. Brooker: “At times, Narcis can be very firm with his staff, especially when they haven't done something just right,” he says, “yet when working with him on school surveys, you see a real gentleness in how he is with children, and a genuine concern for their well-being and to rid them of worms. What Narcis does comes from the heart.”

Dr. Kolackzinski talks highly of Dr. Kabatereine's willingness to share data, to speak frankly at stakeholder meetings, to collaborate with all sorts of people, and to willingly assist other African countries with their NTD control efforts. The Malaria Consortium is currently working with the Government of Southern Sudan on the integrated mapping and control of NTDs, and Dr. Kabatereine has been acting as an adviser. “The team of technicians that Narcis sent to southern Sudan to work with us worked like clockwork,” says Dr. Kolackzinski. “They did all the lab tests for mapping and trained the Sudanese surveyors. They were genuinely committed to training others.” Dr. Kolackzinski credits Narcis's leadership for the technicians' disciplined approach. “Narcis is a useful coach for other African countries,” says Dr. Kolackzinski, “especially for schistosomiasis and soil-transmitted helminths.”

I get a strong sense of Dr. Kabatereine's willingness to speak frankly about controversial issues when I ask him about Uganda's recent move towards integrated control of NTDs, which is being supported by funding from the U.S. Agency for International Development (USAID). Current strategies for controlling several different NTDs, says Dr. Kabatereine, are very similar—they all involve training community medicine distributors (CMDs) and schoolteachers to deliver MDA. So it makes great sense to try and integrate these strategies, he says, and deliver multiple interventions at once. “Reaching the communities themselves within a single period—it's an idea that's quite salient.” Uganda is integrating the control of five diseases: schistosomiasis, soil-transmitted helminths, onchocerciasis, trachoma, and lymphatic filariasis. “None of us want to go back to vertical programs,” he says. “We all want to prove that integration works.”

Nevertheless, he is critical of the way in which USAID has set up a parallel structure, largely outside of the Ministry of Health, to manage the integration. The development agency is channeling funding through the research organization RTI International and Dr. Kabatereine feels that the ministry and the national health system have been sidelined. He worries what will happen when the USAID funding ends—how will integration be sustained if it was never run by the ministry in the first place? “We need strong government involvement in the running,” he says.

Earlier in the day, I had met with Dr. Ambrose Onapa, RTI International's Programme Manager for NTDs, whose office is just down the hallway. He explains that RTI International is providing the funding required by the districts and the ministry of health for implementation of the integrated control program, and is also providing personnel to manage the funds. Implementation is facilitated by an NTD Secretariat at VCD, made up of himself and the individual program managers for each different NTD. Dr. Kabatereine chairs this Secretariat.

“Our role as secretariat is unclear,” says Dr. Kabatereine. “I feel we should be coordinating the program.” In the future, he believes that donors should make sure that the ministry of health has a strong say in the management of integrated control. “Integration isn't easy. It should go directly through the health systems,” he says. He is concerned that CMDs are required to deliver many interventions at once, yet most have little formal education and find it hard to grasp the instructions in the thick integrated training manuals. If this in turn leads to less time spent educating the community about the nature of NTDs, the success of the integrated program could be jeopardized.

Rumors have been going around in the communities, he says, that the medicines are a form of birth control, and such rumors threaten coverage levels. He fears that the problem arises when the CMDs don't give people enough information. He points me to a 2008 social science study on NTDs in North West Uganda, by Melissa Parker and colleagues, which found that adults were increasingly rejecting MDAs through fear of side effects, local beliefs about worms, and inadequate health education [1]. “If you want to integrate,” he says, “you have to be very careful how you train people. The coverage rates will be affected.”

It is rare for public health professionals to be willing to go on the record in criticizing a donor-funded program. My interview with Dr. Kabatereine was part of a fellowship in global health reporting that took me to southern Sudan, Uganda, and Kenya [2], and while many of my interviewees would castigate bilateral or multilateral donor agencies, they would only do so off the record.

So I make double sure that Dr. Kabatereine is happy to be quoted with his concerns about the RTI International program. He confirms that he is. “I don't know what impact I'll face,” he says, but “the story must be told.” He wants to lay out the challenges of integration, not because the idea itself is flawed but because “all these challenges are manageable. We can solve all of these.”

He shares with me an idea that he thinks would go some way to addressing these challenges. The USAID/RTI program is supporting seven African countries to scale up integrated control of NTDs [3]. He urges RTI to bring the ministries of health of all seven countries together in a meeting to share experiences, to learn from each other, and to give them a sense of buy-in. “It would be in the interest of RTI,” he says. “I believe RTI wants to do the best. We would all be winners.”

As our interview ends, he throws out other ideas that he believes would have an impact on Uganda's NTD burden, such as developing anti-parasitic drug formulations specifically for children. Instead of giving children crushed albendazole/mebendazole tablets mixed with dirty water, he says, a practice that can transmit disease, “we should advocate to develop syrups.” And money should be given to health educators at the district level, he argues, to show videos in schools and churches to educate the community about NTDs and their control.

“I hope one day I will succeed with these ideas,” says Dr. Kabatereine. From what I have learned of him, I feel confident he will be a forceful and vocal champion for his suggestions.
